# The Leicester Provident Dispensary.—A Rejoinder

**Published:** 1888-06-09

**Authors:** A. A. Isaacs

**Affiliations:** Christchurch Vicarage, Leicester.


### The Editor's Letter-Box.

[Our Correspondents are reminded that prolixity is a great bar to publication, and that brevity of style and conciseness of statement greatly facilitate early insertion.]

### THE LEICESTER PROVIDENT DISPENSARY.—A REJOINDER.

Sir,—I have just received a copy of your journal of this date, which contains a letter from Mr. M. Maxfield, of this town, purporting to be a rejoinder to some comments which I presume you had made on a former occasion concerning the management of the Leicester Provident Dispensary. At the annual meeting on March 14th, the Board, supported by the majority of subscribers present, refused to sanction a fair and full inquiry into the grave charges made against the management. With delightful complaisance they expressed their willingness to hear and consider these charges themselves, and to adjudicate on the matters in which they are themselves the accused. Twenty-six of the leading medical men of the town had signed a declaration that "essential changes" are necessary in the management of the institution, of whom three are on the present staff of the Dispensary, and three have formerly been so. Of the other members of the existing medical staff, I am told that they could find but one who would venture to exonerate the management. Collectors are employed in touting for members, and because the medical profession generally protest against the large number of persons (adding to the percentage of the manager) who are thus led to enter the Dispensary, whose ample means should disqualify them for membership, Mr. Maxfield denounces them as enemies of the institution. We feel that in a *bona fide* inquiry the medical profession should be represented, but be that as it may, we are determined that this shall be done in such a form as to command the confidence of the members and the public. The guilty alone have need to dread a full and exhaustive investigation. At the annual meeting I pointed out that the institution began the year with a debt of nearly £500, and that although legacies and donations had been received to the amount of £435, the debt had only been reduced by £76. The state of inexcusable and unaccountable indebtedness is chronic. Is it not simply infamous that, having, as Mr. Maxfield says, "over thirty-five thousand members" (by whom it ought to be more than self-supporting), such a state of things should exist? It is this (mis) management which is paraded before the public as successful and inimitable!

Christchurch Vicarage, Leicester. A. A. Isaacs.

